# Underutilization and disparities in access to *EGFR* testing among Medicare patients with lung cancer from 2010 – 2013

**DOI:** 10.1186/s12885-018-4190-3

**Published:** 2018-03-20

**Authors:** Julie A. Lynch, Brygida Berse, Merry Rabb, Paul Mosquin, Rob Chew, Suzanne L. West, Nicole Coomer, Daniel Becker, John Kautter

**Affiliations:** 1 Department of Veterans Affairs Salt Lake City Health Care System, Salt Lake City, UT USA; 2 RTI International Waltham, Waltham, MA USA; 3 Boston University School of Medicine, Boston, MA USA; 4 RTI International, Research Triangle Park, Durham, NC USA; 5 Veterans Health Administration, New York, NY USA; 6 New York University, New York, NY USA; 7 Veterans Health Administration, 200 Springs Road, Building 70, Bedford, MA 01730 USA

**Keywords:** Lung cancer, *EGFR* testing, Medicare, Hospital referral region, Billing code

## Abstract

**Background:**

Tumor testing for mutations in the epidermal growth factor receptor (EGFR) gene is indicated for all newly diagnosed, metastatic lung cancer patients, who may be candidates for first-line treatment with an *EGFR* tyrosine kinase inhibitor. Few studies have analyzed population-level testing.

**Methods:**

We identified clinical, demographic, and regional predictors of *EGFR & KRAS* testing among Medicare beneficiaries with a new diagnosis of lung cancer in 2011–2013 claims. The outcome variable was whether the patient underwent molecular, *EGFR* and *KRAS* testing. Independent variables included: patient demographics, Medicaid status, clinical characteristics, and region where the patient lived. We performed multivariate logistic regression to identify factors that predicted testing.

**Results:**

From 2011 to 2013, there was a 19.7% increase in the rate of *EGFR* testing. Patient zip code had the greatest impact on odds to undergo testing; for example, patients who lived in the Boston, Massachusetts hospital referral region were the most likely to be tested (odds ratio (OR) of 4.94, with a 95% confidence interval (CI) of 1.67–14.62). Patient demographics also impacted odds to be tested. Asian/Pacific Islanders were most likely to be tested (OR 1.63, CI 1.53–1.79). Minorities and Medicaid patients were less likely to be tested. Medicaid recipients had an OR of 0.74 (CI 0.72–0.77). Hispanics and Blacks were also less likely to be tested (OR 0.97, CI 0.78–0.99 and 0.95, CI 0.92–0.99), respectively. Clinical procedures were also correlated with testing. Patients who underwent transcatheter biopsies were 2.54 times more likely to be tested (CI 2.49–2.60) than those who did not undergo this type of biopsy.

**Conclusions:**

Despite an overall increase in *EGFR* testing, there is widespread underutilization of guideline-recommended testing. We observed racial, income, and regional disparities in testing. Precision medicine has increased the complexity of cancer diagnosis and treatment. Targeted interventions and clinical decision support tools are needed to ensure that all patients are benefitting from advances in precision medicine. Without such interventions, precision medicine may exacerbate racial disparities in cancer care and health outcomes.

**Electronic supplementary material:**

The online version of this article (10.1186/s12885-018-4190-3) contains supplementary material, which is available to authorized users.

## Background

In 2017, approximately 222,500 patients within the United States (U.S.) will be diagnosed with lung cancer and 155,870 are expected to die from it [1]. The average age at lung cancer diagnosis is 70 years, and 68% of patients are 65 years or older and eligible for Medicare [2]. Lung cancer causes serious medical problems or death in 1.7% of Medicare beneficiaries [3]. Studying lung cancer molecular test utilization within Medicare claims provides a unique opportunity for a comprehensive, population-level analysis of precision medicine testing.

Over the last decade, molecular testing of lung tumors has become an essential component of diagnosis and treatment of advanced non-small cell lung cancer (NSCLC). Molecular testing facilitates targeted treatment directed at specific genetic alterations in tumors [4]. There are now multiple drugs approved by the Food & Drug Administration (FDA) to treat lung cancer patients with specific tumor alterations (see Additional file 1).

Mutations of the epidermal growth factor receptor (*EGFR)* gene and chromosomal rearrangement of the anaplastic lymphoma kinase (*ALK*) gene were among the first established molecular targets for therapy in lung adenocarcinoma. Testing for these two markers identifies a subset of patients for whom specific oral tyrosine kinase inhibitors (TKIs) are most effective. Because *KRAS* and *EGFR* mutations are mutually exclusive in greater than 99% of cases, analysis of *KRAS* mutational status has also been used to exclude sensitivity to *EGFR* inhibitors [5]. However, guidelines do not recommend use of *KRAS* testing in lung cancer.

Guidelines for *EGFR* testing and targeted treatment evolved gradually over the past decade, as illustrated in Additional file 2. The FDA approved erlotinib in 2004 for second-line therapy regardless of tumor histologic type or *EGFR* status. It was not until 2011, however, that clinical guidelines linked erlotinib treatment with *EGFR* testing [6–9]. From 2011 through 2013, *EGFR* testing was indicated for all patients with newly diagnosed metastatic adenocarcinoma of the lung being considered for first-line therapy with an *EGFR* tyrosine kinase inhibitor. This indication corresponded to approximately 35% of all new lung cancer cases [10, 11]. *EGFR* testing was also recommended for patients with recurrent metastatic disease. In 2013, FDA approved erlotinib as first line therapy for *EGFR* mutation-positive patients. According to Local Coverage Determinations by several Medicare contractors, reimbursement for the *EGFR* test applies to patients with clinical indications for testing [12].

The importance of *EGFR* testing for diagnosis and treatment of lung cancer is illustrated by its worldwide availability. *EGFR* testing is now available in 57 countries [13]. Test availability and cost are strongly correlated with the Human Development Index of a given country, underscoring the importance of bringing precision medicine to underserved populations.

The prevalence of *EGFR* mutations in lung adenocarcinomas differs by patient ancestry. Among patients of European descent, mutation prevalence is between 10% to 15%, and among Asians it can be as high as 50%, with higher frequency in women and non-smokers across all ethnicities [5]. Within populations, the *EGFR* mutation rate may differ among specific patient groups based on their history of smoking. Among U.S. veterans, mutation prevalence is lower (7%), likely due to the high number of veterans who smoke [11]. *EGFR* mutations are less frequent in squamous cell carcinomas therefore the guidelines suggest testing only those patients with squamous histology whose clinical or demographic characteristics (e.g., absence of smoking history, Asian descent) indicate an increased likelihood of mutations.

Assays used to detect *EGFR* mutations can be limited by the amount of tissue available from the biopsy specimen. Thus, patient-level differences in testing may be partially explained by the types of diagnostic procedures patients undergo. Two decades of research have demonstrated racial, regional, and socioeconomic differences in access to lung cancer surgical procedures [14–21]. Black patients and those of low socioeconomic status were less likely to receive surgery or biopsies that yield enough lung tumor tissue for identifying *EGFR* mutations. Disparities in treatment and outcomes of lung cancer were most pronounced among Black men, who are diagnosed an average of four years younger than Whites and who experienced a significantly lower five-year survival rate [22].

There were four objectives of this study: (1) Identify Medicare patients with newly diagnosed lung cancer who underwent molecular and proteomic testing. (2) Compare the proportion of patients tested to the expected rate of testing based on population-level statistics reported in cancer-registry data. (3) Identify patient-level and regional variations in access to *EGFR and KRAS* testing; and (4) Evaluate whether patient-level disparities in access to diagnostic procedures compound disparities in access to *EGFR* testing.

## Methods

### Data sources

We conducted a retrospective study using secondary data analysis methods. The primary data source was Medicare claims, including 2010–2013 100% inpatient MedPAR, Part B, and Outpatient files. Additional data sources included the Denominator file (100%), the Hierarchical Condition Categories (HCC) risk score file, Provider of Service file, Health Resources and Services Administration Area Health Resource File [23] and the Dartmouth Atlas Hospital Referral Region (HRR) database [24].

Our analytic sample consisted of patients who met the following criteria:

1. Were Medicare fee-for-service beneficiaries who had a diagnosis code for lung cancer (malignant neoplasm of trachea, bronchus, and lung, (International Classification of Disease (ICD-9) 162.0–162.9) in any diagnosis fields within MedPAR, Outpatient, or Part B files.
2. Had a short term or specialty hospital encounter in a physician office, inpatient or outpatient hospital, or ambulatory surgical center.
3. Sought lung cancer treatment from 2010 to 2013, defined as having a lung biopsy and/or a lung surgery, or lung surgical pathology analysis (ICD-9-CM and Current Procedural Terminology (CPT)/Healthcare Common Procedure Coding System (HCPCS) codes in Table 3) claim in MedPAR, outpatient or Part B files.

We restricted our analysis to claims that had lung cancer listed as the line item or principal diagnosis code. MedPAR, outpatient, and Part B claims were rolled up to a patient-level analytic file that became the basis for the study. We used a validated algorithm to identify incident lung cancer cases in Medicare claims data [25–27]. This algorithm relied on identifying newly diagnosed cases by restricting analysis to patients who had a new claim for lung cancer diagnosis and twelve months of claims history without a previous indication of lung cancer. Most patients with lung cancer are diagnosed at an advanced stage of the disease, when symptoms have progressed and healthcare interventions are essential, which generates continuity in claims prior to and following a diagnosis of lung cancer.

### Variables

The unit of observation was the patient. The outcome variable was whether the patient had a claim for a lung cancer molecular test. We created variables that allowed us to conduct analysis on the total population and a subset of the population. We identified the year of the first lung cancer claim and we identified whether a patient had a year of prior claims data. (Without access to 2009 claims, we had no method for differentiating incident versus prevalent cases in the 2010 claims data. Therefore, we reported molecular test claims for patients identified in 2010 but then dropped these patients from subsequent analysis.) The vast majority (93%) of patients identified in 2011–2013 claims represented newly diagnosed cases.

#### Lung cancer clinical procedures

Using the set of CPT codes listed in Table 3, we created dichotomous variables to identify whether a patient underwent specific lung cancer surgical procedures. Some of these procedures, such as fine-needle aspiration, are unlikely to general sufficient tumor cells for molecular testing. Patients who had a claim for surgical pathology procedures billed with CPT codes 88305, 88307, 88309, with a primary or line item diagnosis code of lung cancer, likely had sufficient lung tumor tissue for testing. These variables allowed us to restrict some analysis to only those patients who underwent a lung biopsy.

Patients who had a lung cancer molecular test were identified using different CPT codes, depending upon the year the claim was submitted. Years 2010–2012, molecular tests were billed with stacked methodology-based codes, which made it impossible to conclusively identify or distinguish *EGFR* or *KRAS* tests from other molecular tests. However, these were essentially the only genetic tests used for lung cancer during this time. Therefore, we identified patients who had a claim for code 83912 (Genetic examination), with line item diagnosis of lung cancer. This code was billed once per procedure and it was included in the stacks of codes used by major reference laboratories for *EGFR* and *KRAS* tests. Code 83912 was discontinued in 2013 and replaced by gene-specific CPT codes. For 2013 claims, we identified patients who had either an *EGFR* (81235) test or a *KRAS* codons 12 and 13 (81275) test. We also identified whether the patient had undergone a proprietary lung cancer proteomic test. This test was billed using a combination of the CPT code 84999 and the Clinical Laboratory Improvement Amendment (CLIA) number (06D1090464) for the laboratory that conducts the test (Biodesix Inc., Boulder, CO).

We also captured variables to identify patient demographics (age, gender, and race), Medicaid status, risk score, zip code of residence, distance to a National Cancer Institute (NCI) designated cancer center, and the HRR in which the patient lived.

### Statistical analysis

We conducted univariate and bivariate analyses, including *t*-tests for continuous variables and χ2 for categorical variables. Statistically significant explanatory variables (*P* values < 0.05) were then included in logistic regression modeling. Statistical analysis was performed using Stata software (version 12.0; StataCorp, College Station, TX).

## Results

### Molecular testing from 2010 to 2013

We identified 1,178,293 Medicare beneficiaries who had a diagnosis code of lung cancer from 2010 to 2013. Among these patients, 62,955 (5%) underwent a molecular test over during that time period. There were 42,415 tests billed using 83912, 18,898 *EGFR* tests, and 8,066 *KRAS* tests. Patients identified in 2010 represented both incident and prevalent cases. Among patients identified with lung cancer in 2010, there were a total of 21,422 who received a molecular test from 2010 through 2013. Most patients (18,845) underwent a test billed with code 83912. There were 2,516 patients who underwent an *EGFR* test and 1,095 who underwent a *KRAS* test. There were 1,034 patients who had claims for multiple molecular tests. Patients identified in 2011 through 2013 represented mostly incident cases. There were 13,568 patients identified in 2011 and 14,302 patients identified in 2012 who underwent at least one molecular test. There were 12,433 patients identified in 2013 who underwent *EGFR* testing; 4,856 who underwent *KRAS* testing; and 3,626 who underwent both *EGFR* and *KRAS* testing.

All subsequent analysis was conducted on patients identified in 2011 through 2013. Table 1 illustrates the percentage of patients tested among newly diagnosed patients who had lung tissue available for analysis. There was a 19.7% increase testing from 2011 through 2013. In 2011, 7.8% of patients who underwent surgical pathology were tested. In 2013, this increased to 9.3%. The absolute number of claims for molecular testing decreased slightly in 2013. This decrease may be explained by limitations in the data. Patients diagnosed in December of 2013 may have been tested in January 2014 or there may be an expansion of next generation sequencing, which would not be billed with the gene-specific billing codes.

**Table 1:** Claims for lung cancer molecular testing among all Medicare beneficiaries diagnosed with lung cancer

By year and billing code					
Year diagnosed	Claim for reporting a molecular test^a^				
	Molecular test 83912	*EGFR*	*KRAS*	Multiple	Total^b^
2010	18,845	2,516	1,095	1,034	21,422
2011	12,254	1,235	655	576	13,568
2012	11,316	2,714	1,460	1,188	14,302
2013	–	12,433	4,856	3,626	13,663
Total	42,415	18,898	8,066	6,424	62,955
Percent of patients tested					
		Years			
		2011	2012	2013	Change 2011–2013
Patients diagnosed with lung cancer		245,576	227,929	215,036	−12.4%
Patients who a claim for surgical pathology analysis^c^		167,291	155,408	142,469	−14.8%
Patients who had a claim for a molecular test^d^		13,008	13,818	13,259	1.9%
Percent of patients with tissue who were tested		7.78	8.89	9.31	19.7%

^a^Current Procedural Terminology (CPT)/ Healthcare Common Procedure Coding System (HCPCS) codes 83912 for years 2011–2012; 81235 (EGFR) and 81275 (KRAS) for 2013

^b^Total represents patients who had claims for 83912, 81235, and 81275

^c^Current Procedural Terminology (CPT)/ Healthcare Common Procedure Coding System (HCPCS) codes 88305, 88307, 88309

^d^CPT/HCPCS codes 83912 for years 2011–2012; 81235 and 81275 for 2013

### Patient characteristics by lung tissue analysis and molecular testing

Lung cancer diagnosis was associated with increasing age. In the overall Medicare population, 37% of beneficiaries are 75 years or older [28]. In our analysis, 298,829 (43.4%) of beneficiaries were 75 or older and the mean age was 72.9 (standard deviation (SD) 9.5). Lung cancer diagnosis was more common in White patients than among other racial groups. Whites represented 80.8% of all Medicare patients [28] but 85.1% of the cohort we examined. In contrast, Asian patients represented 2.1% of Medicare beneficiaries but only 1.5% of lung cancer patients.

Table 2 illustrates a bivariate analysis of patients by whether they had a claim for surgical pathology analysis and molecular testing. Among patients identified from 2011 through 2013, there were 465,168 (67.6%) who underwent surgical pathology analysis of lung tissue and 40,085 (8.6%) of those patients underwent a molecular test.

**Table 2:** Demographics of Lung Cancer Patients in Medicare Who Underwent Surgical Pathology Analysis and Molecular Testing for Lung Cancer in from 2011 to 2013

**Total newly diagnosed patients 2011–2013**						
	2011–2013					
	Newly identified patients	Surgical pathology^a^	Molecular test^b^	Percent with surgical pathology	Percent tested	
Total	688,541	465,168	40,085	67.6	8.6	
Newly diagnosed	642,570	443,483	38,170	69.0	8.6	
Demographics characteristics						
Age group						
0–54	24,567	15,831	941	64.4	5.9	
55–59	24,598	15,772	1,046	64.1	6.6	
60–64	59,883	35,144	2,842	58.7	8.1	
65–69	141,033	96,945	9,347	68.7	9.6	
70–74	137,641	97,741	9,300	71.0	9.5	
75+	298,829	203,120	16,571	68.0	8.2	
Missing	1,990	615	38	30.9	6.2	
Gender						
Male	363,596	249,182	19,707	68.5	7.9	
Female	322,955	215,371	20,340	66.7	9.4	
Missing	1,990	615	38	30.9	6.2	
Race/Ethnicity						
White	586,000	401,995	34,808	68.6	8.7	
Black	67,982	41,922	3,071	61.7	7.3	
Asian/Pacific Islander	10,488	6,758	929	64.4	13.7	
Hispanic	8,580	5,301	342	61.8	6.5	
North American/Native	2,722	1,936	137	71.1	7.1	
Other, unknown, or missing	12,769	7,256	798	56.8	11.0	
Medicaid Status						
Medicaid	141,724	94,771	6,324	66.9	6.7	
No Medicaid	544,827	369,782	33,723	67.9	9.1	
Missing	1,990	615	38	30.9	6.2	
Clinical characteristics						
Risk score (Mean/SD)					–	
Less than or equal 1	322,995	227,476	24,141	70.4	10.6	
Between 1.0–1.9	194,281	129,935	10,079	66.9	7.8	
Greater than 1.9 (mean)	171,265	107,757	5,865	62.9	5.4	
Regional characteristics						
Distance to an NCI CC (Mean (SD) in miles)	85.5 (111.4)	86.5 (107.7)	82.4 (103.6)			
Metropolitan county	546,710	362,381	32,100	66.3	8.9	
Nonmetropolitan county	141,831	102,787	7,985	72.5	7.8	
Newly diagnosed patients per year						
	2011			2012		
	Surgical pathology	Molecular test	Percent tested	Surgical pathology	Molecular test	Percent tested
Total	167,291	13,008	7.8	155,408	13,818	8.9
Newly diagnosed	158,654	12,256	7.7	147,459	13,120	8.9
Demographics characteristics						
Age group						
0–54	5,955	312	5.2	5,404	314	5.8
55–59	5,628	323	5.7	5,294	374	7.1
60–64	13,058	985	7.5	12,354	1,045	8.5
65–69	34,591	3,085	8.9	32,582	3,217	9.9
70–74	34,958	3,010	8.6	32,057	3,133	9.8
75+	72,869	5,278	7.2	67,500	5,727	8.5
Missing	232	15	6.5	217	8	3.7
Gender						
Male	88,082	6,193	7.0	83,098	6,804	8.2
Female	78,977	6,800	8.6	72,093	7,006	9.7
Missing	232	15	6.5	217	8	3.7
Race/Ethnicity						
White	144,973	11,342	7.8	134,110	11,969	8.9
Black	14,973	936	6.3	14,206	1,093	7.7
Asian/Pacific Islander	2,336	330	14.1	2,293	313	13.7
Hispanic	1,961	105	5.4	1,739	123	7.1
North American/Native	665	46	6.9	636	42	6.6
Other, unknown, or missing	2,383	249	10.4	2,424	278	11.5
Medicaid Status						
Medicaid	33,488	1,988	5.9	32,024	2,238	7.0
No Medicaid	133,571	11,005	8.2	123,167	11,572	9.4
Missing	232	15	6.5	217	8	3.7
Clinical characteristics						
Risk score (Mean/SD)	1.4 (1.1)	1.1 (0.9)	–	1.4 (1.2)	1.1 (0.9)	–
Less than or equal 1	79,620	7,717	9.7	74,947	8,242	11.0
Between 1.0–1.9	47,962	3,414	7.1	43,333	3,642	8.4
Greater than 1.9 (mean)	39,709	1,877	4.7	37,128	1,934	5.2
Regional characteristics						
Distance to an NCI CC (Mean (SD) in miles)	86.5 (108.5)	79.4 (104.8)		86.2 (107.3)	83.0 (101.5)	
Metropolitan county	130,669	10,526	8.1	121,214	11,110	9.2
Nonmetropolitan county	36,622	2,482	6.8	34,194	2,708	7.9
**Newly diagnosed patients per year**						
	2013					
	Surgical pathology	EGFR	KRAS	Both	Total tested	Percent tested
Total	142,469	12,090	4,677	3,514	13,250	9.3
Newly diagnosed	137,370	11,661	4,514	3,381	12,794	9.3
Demographics characteristics						
Age group						
0–54	4,472	271	107	63	315	7.0
55–59	4,850	311	128	90	349	7.2
60–64	9,732	721	294	203	812	8.3
65–69	29,772	2,781	1,122	858	3,045	10.2
70–74	30,726	2,887	1,123	853	3,157	10.3
75+	62,751	5,105	1,895	1,434	5,566	8.9
Missing	166	14	5	13	6	3.6
Gender						
Male	78,002	6,044	2,447	1,781	6710	8.6
Female	64,301	6,032	2,222	1,720	6,534	10.2
Missing	166	14	5	13	6	3.6
Race/Ethnicity						
White	122,912	10,513	4,036	3,052	11,497	9.4
Black	12,743	933	352	243	1,042	8.2
Asian/Pacific Islander	2,129	262	117	93	286	13.4
Hispanic	1,601	98	46	30	114	7.1
North American/Native	635	45	15	11	49	7.7
Other, unknown, or missing	2,449	239	108	85	262	10.7
Medicaid Status						
Medicaid	29,259	1,885	722	509	2,098	7.2
No Medicaid	113,044	10,191	3,947	2,992	11,146	9.9
Missing	166	14	5	13	6	3.6
Clinical characteristics						
Risk score (Mean/SD)	1.2 (1.0)	1.0 (0.8)	1.0 (0.9)	1.0 (0.8)	1.0 (0.8)	–
Less than or equal 1	72,909	7,501	2,883	2,202	8,182	11.2
Between 1.0–1.9	38,640	2,783	1,028	788	3,023	7.8
Greater than 1.9 (mean)	30,920	1,806	763	515	2,054	6.6
Regional characteristics						
Distance to an NCI CC (Mean (SD) in miles)	86.8 (107.2)	85.3 (106.2)	69.0 (78.25)	66.1 (74.9)	84.6 (104.6)	
Metropolitan county	110,498	9,541	3,832	2,918	10,455	
Nonmetropolitan county	31,971	2,549	845	596	2,798	

Source: RTI analysis of 2011–2013 Medicare

^a^ As identified by HCPCS codes 88305, 88307

^b^ As identified by HCPCS code 83912, 81235

There was essentially no difference in the age across years or by sugical pathology or testing status. Mean age was 72.9 with SD of 9.5

We observed significant racial differences in percentage of patients who underwent lung tissue analysis. Among North American Natives and Whites, 71% and 68.6%, respectively, had claims for surgical pathology analysis. Only 61.7% of Blacks had claims for lung tissue analysis. Identifying patient-level differences in access to surgical pathology is important because these patients will not have access to lung tissue molecular testing. Further, the denominator in the analysis of molecular testing was restricted to patients who had lung tissue available for analysis.

There were small but statistically significant differences by age, race, Medicaid status, and risk score and testing. Beneficiaries under age 55 were the least likely to be tested (5.9%), which may be explained by an earlier stage of diagnosis or by these patients being diagnosed and tested prior to enrolling in Medicare. Among racial/ethnic groups, a greater percentage of Asian/Pacific Islanders were tested (13.7%) compared to Whites (8.7%), Blacks (7.3%), Hispanics (6.5%), and North American Natives (7.1%). A greater percentage of non-Medicaid patients were tested than patients who received Medicaid (9.1% compared to 6.7%). Testing was also associated with patient comorbidity, as measured by the HCC risk score. The mean HCC risk score for all Medicare beneficiaries is 1. The mean risk score for lung cancer patients is 1.9. Lung cancer patients with low risk scores (below 1) were twice as likely to be tested compared to those with risk scores above the mean risk score (10.6% vs. 5.4%, respectively).

There were also regional differences in access to molecular testing. Testing was associated with living in closer proximity to an NCI designated cancer center and in a metropolitan county. Patients tested lived an average of 4 miles closer to an NCI cancer center than a non-tested patient. In 2013, the relationship between testing status and distance to an NCI cancer center was much stronger for *KRAS* testing than for *EGFR* testing. Patients undergoing both *KRAS* and *EGFR* testing lived an average of 66 miles away from an NCI cancer center compared to 85 miles for those undergoing *EGFR* testing.

### Regional differences in lung cancer molecular testing

**Fig. 1:**
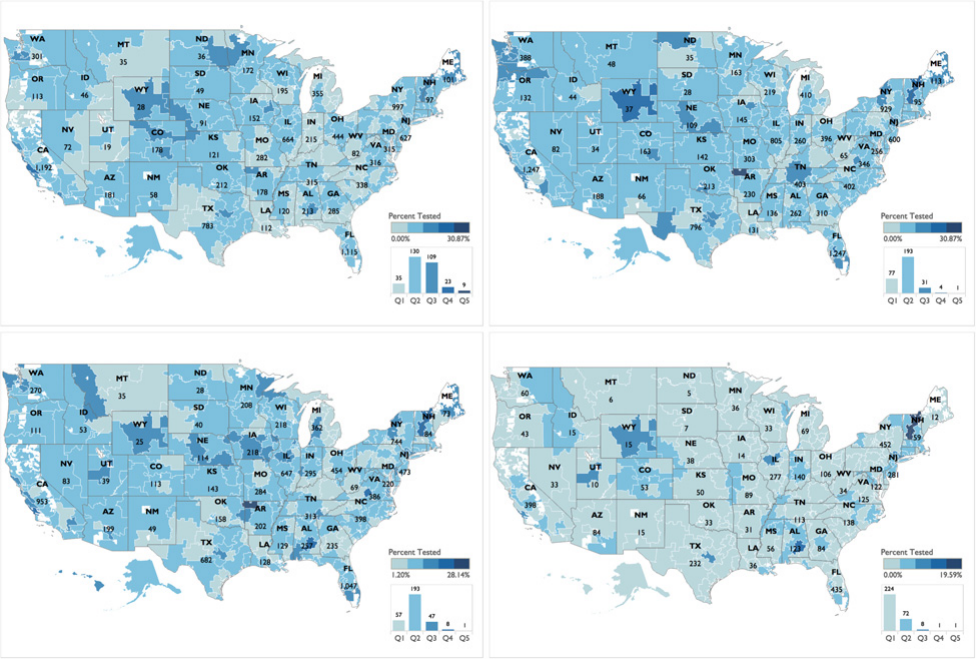
Molecular testing by HRR and state. **a**. Upper left – Molecular test (code 83912) in 2011. **b.** Upper right - Molecular test (code 83912) in 2012. **c.** Lower left - EGFR testing (code 81235) in 2013. **d.** Lower right – *KRAS* testing (code 81275) in 2013

We analyzed the variability of testing across states and HRRs (See Additional file 3). The latter are areas served by individual referral centers [24]. Most Americans seek care from hospitals that are near their place of residence [24], therefore the beneficiaries’ zip codes provide statistically reliable information about the HRR. The upper left quadrant of Fig. 1 illustrates the percentage of patients in 2011, by HRR and by state, who had a claim for lung tissue surgical pathology and who underwent molecular testing. Testing by HRR ranged from no patients tested in Mason City, Iowa to 18.86% of patients testing in Salinas, California. Colorado was the state with the highest percentage of patients tested with 188 claims for lung cancer molecular tests, (12.40%) of patients who underwent lung tissue surgical pathology analysis. Utah had the lowest percentage of patients tested (4.23% or 19 tests). The upper right quadrant of Fig. 1 illustrates that in 2012 there was a 1% increase in percentage of patients tested, with some patients in every HRR being tested. In 2012, the range of patients tested was from 2.15% of patients testing in Rochester, Minnesota to 30.86% in Springdale, Arkansas. Wyoming had the highest percentage of patients tested (15.9% or 37 tests). Puerto Rico had the lowest percentage of patients tested (3.4% or 7 tests). In 2013, introduction of the gene specific CPT codes allowed us to distinguish between *EGFR* resting and *KRAS* testing. Figure 1c and d illustrate the percentage of patients in 2013 who underwent *EGFR* and *KRAS* testing, respectively. The overall percentage of patients who underwent *EGFR* testing was 8.5%, which was a decrease from 2012 but represents specific *EGFR* testing. The greatest percentage of patients tested (29%) remained those living in Springdale, Arkansas. The HRR with the lowest percentage of patients tested was in Covington, Kentucky (1.2%). The state with the highest percentage of patients who underwent *EGFR* testing was Vermont (18.9%, 44 tests). As discussed previously, *KRAS* testing was closely associated with proximity to NCI cancer centers. The highest percentage of patients who underwent *KRAS* testing was in Montgomery, Alabama (13.4%, 28 patients tested).

### Relationship between testing and type of biopsy or lung surgical procedures

We then analyzed whether there was a relationship between testing and type of surgical procedure. These results are presented in Table 3. This analysis was restricted to those patients who underwent lung tissue analysis as indicated by codes 88305, 88307, and 88309. There were differences in the type of lung surgery performed and testing status. Among procedures that yield tumor tissue, the percentage of patients tested was highest among those who underwent video-assisted thoracoscopic surgery (16.9% of patients tested) followed by mediastinoscopy (16.6% of patients tested). The data point to a correlation between undergoing other types of surgical procedures and testing. Although a thoracentesis does not yield tumor tissue, the percentage of patients tested among those who underwent this procedure was still greater than among all patients with claims for surgical pathology (10.3% vs. 8.6%). However, these categories are not exclusive. Therefore, patients who underwent thoracentesis may have also had another procedure in which lung tissue was analyzed.

**Table 3:** HCPCS and ICD9 procedure codes used to identify lung biopsies and surgeries in the Medicare Fee-for-Service Part B claims

Procedure	HCPCS codes	Surgical pathology	Molecular test	Percent tested
Patients who underwent tissue analysis	88305, 88307, 88309	465,168	40,085	8.6
Fine-needle aspiration	10021, 10022	48,901	5,789	11.8
Lymph node biopsy	38505	8,741	957	10.9
Bronchoscopy	31622, 31623, 31624, 31625, 31626, 31627	143,900	16,608	11.5
Thoracentesis	32421	25,854	2,675	10.3
Endobronchial ultrasound	31620	34,512	5,232	15.2
Transbronchial needle aspiration	31628, 31629, 31630, 31631, 31632, 31633	79,480	10,415	13.1
Other bronchoscopies	31635, 31636, 31637, 31638, 31640, 31641, 31643, 31645, 31646	16,365	1,668	10.2
Transcatheter biopsy	32400, 32405	130,485	19,063	14.6
Mediastinoscopy	39400	21,654	3,603	16.6
Video-assisted thoracoscopic surgery	32663, 32665, 32666, 32667, 32668, 32669, 32670, 32671, 32672	24,739	4,173	16.9
Open surgery	32440, 32442, 32445, 32480, 32482, 32484, 32488, 32491, 32505, 32506, 32507	32,886	4,778	14.5
Thoracotomy	32,096, 32097, 32098, 32110, 32120, 32124, 32140, 32141, 32486, 32501	5,594	704	12.6
Thoracoscopy	32601, 32602, 32603, 32604, 32605, 32606, 32650, 2651, 32652, 32653, 32654, 32655, 32656, 32657, 32658, 32659, 32660, 32661, 32662, 32663	34,496	5,315	15.4

Abbreviations: *HCPCS* Healthcare Common Procedure Coding System, *ICD-9 CM* International Classification of Disease

Source: RTI analysis of 2011–2013 Medicare claims data

### Factors that predict utilization of lung cancer molecular testing

The relationship between patient characteristics and odds to undergo testing persisted in multivariate logistic regression analysis (Table 4). A patient’s address had the greatest impact on testing status. Patients who lived in the Boston, Massachusetts HRR had an odds ratio (OR) of 4.94, with a 95% confidence interval (CI) of 1.67–14.62. Patients living in the Los Angeles, CA HRR were equally likely to be tested (OR 4.94, CI 2.08–11.71). Patients least likely to be tested lived in the Mason, Indiana HRR (OR 0.10, CI .0.4–0.30).

**Table 4:** Characteristics that predict use of molecular tests among lung cancer patients

Variable	OR	*P* Value	95% CI
Demographic characteristics			
Age (per year)	0.99	0.00	0.99–0.99
Asian (vs. White)	1.63	0.00	1.53–1.79
Black (vs. Whites)	0.95	0.05	0.92–0.99
Hispanic (vs. Whites)	0.87	0.03	0.78–0.99
Female (vs. Whites)	1.18	0.00	1.16–1.21
Medicaid recipient (vs. all others)	0.74	0.00	0.72–0.77
Distance to NCI Cancer Center (per mile)	0.99	0.00	0.99–0.99
Number of lung cancers patient in HRR	0.99	0.01	0.99–0.99
Clinical characteristics (vs. all others)			
Transcatheter biopsy	2.54	0.00	2.49–2.60
TBNA	1.53	0.00	1.48–1.59
Inpatient stay	1.48	0.00	1.45–1.51
Thoracoscopy	1.42	0.00	1.36–1.48
Mediastinoscopy	1.35	0.00	1.30–1.41
EBUS	1.32	0.00	1.27–1.38
Bronchoscopy	1.24	0.00	1.21–1.27
Open surgery	1.17	0.00	1.13–1.22
VATS	1.14	0.00	1.09–1.20
Year identified	1.09	0.00	1.09–1.12
HCC risk score	0.77	0.00	0.76–0.78
Regional characteristics			
Distance to NCI Cancer Center	0.99	0.00	0.99–0.99
Number of lung cancer patient in HRR	0.99	0.01	0.99–0.99
20 HRRs with increased likelihood of testing			
Boston, MA	4.94	0.00	1.67–14.62
Los Angeles, CA	4.94	0.00	2.08–11.71
East Long Island, NY	4.25	0.00	1.92–9.43
Manhattan, NY	2.99	0.00	1.69–5.31
Fort Lauderdale, FL	2.89	0.00	2.12–3.95
Philadelphia, PA	2.67	0.00	1.50–4.76
Camden, NJ	2.57	0.00	1.48–4.48
Orlando, FL	2.25	0.01	1.21–4.20
Anchorage, AK	2.21	0.00	1.29–3.77
Springdale, AR	2.08	0.03	1.09–4.00
Houston, TX	1.93	0.01	1.14–3.24
Atlanta, GA	1.83	0.04	1.02–3.30
St. Louis, MO	1.71	0.03	1.04–2.79
Pittsburgh, PA	1.66	0.01	1.13–2.43
Nashville, TN	1.59	0.00	1.31–1.93
Miami, FL	1.49	0.00	1.23–1.80
Columbus, OH	1.44	0.00	1.17–1.76
Seattle, WA	1.39	0.01	1.09–1.76
Fort Myers, FL	1.37	0.03	1.03–1.82
Kansas City, MO	1.32	0.00	1.12–1.55
20 HRRs with lowest likelihood of testing			
Cape Girardeau, MO	0.26	0.00	0.12–0.56
Binghamton, NY	0.25	0.00	0.12–0.54
St. Cloud, MN	0.25	0.00	0.10–0.61
La Crosse, WI	0.24	0.00	0.10–0.57
Corpus Christi, TX	0.24	0.00	0.11–0.51
San Angelo, TX	0.24	0.00	0.09–0.59
Abilene, TX	0.23	0.00	0.10–0.51
Covington, KY	0.23	0.00	0.10–0.50
Longview, TX	0.22	0.00	0.09–0.53
Slidell, LA	0.21	0.00	0.09–0.53
Grand Forks, ND	0.21	0.00	0.09–0.53
Tuscaloosa, AL	0.21	0.00	0.09–0.48
Wichita Falls, TX	0.20	0.00	0.08–0.47
Sayre, PA	0.18	0.00	0.07–0.46
Texarkana, AR	0.16	0.00	0.07–0.38
Idaho Falls, ID	0.16	0.00	0.05–0.48
Alexandria, LA	0.15	0.00	0.06–0.34
Rochester, MN	0.15	0.00	0.06–0.35
Rome, GA	0.12	0.00	0.05–0.28
Mason City, IA	0.10	0.00	0.04–0.30

Abbreviations: VS versus, *OR* Odds ratio, *CI* Confidence Interval, *HCC* hierarchical condition categories, *HRR* Hospital referral regions, *TBNA* transbronchial needle aspiration, *VATS* video-assisted thoracic surgery, *EBUS* endobronchial ultrasound

Source: RTI analysis of 2011–2013 Medicare claims data

Reference groups: Race/ethnic groups, Medicaid status, and clinical characteristics were dichotomous variables

Reference group for HRR is Birmingham, Alabama which had the median percentage of patients tested (8%)

Clinical procedures had the next strongest correlation with testing. Patients who had a transcatheter biopsy were 2.54 times more likely to be tested (CI 2.49–2.60) than those who did not undergo this type of biopsy. This was followed by patients who had a transbronchial needle aspiration (TNBA, OR 1.53, CI 1.48–1.59). There was also a significant increase in odds to be tested by year diagnosed. Patients diagnosed in 2012 were more likely to be tested than patients diagnosed in 2011 (OR 1.09, CI 1.09–1.12). As patients’ level of comorbidities increased, their odds to be tested decreased. To interpret the OR of continuous variables such as HCC risk score (which is measured in increments of 0.001), we obtained the logit coefficient and multiplied by a factor. There was a 23% decreased odds of testing for a 1 unit increase in risk score.

Patient demographics also had an impact on likelihood to be tested. Asian/Pacific Islanders were most likely to be tested (OR 1.63, CI 1.53–1.79). Minorities and Medicaid patients were less likely to be tested. Medicaid recipients had an OR of 0.74 (CI 0.72–0.77). Hispanics and Blacks were also less likely to be tested (OR 0.97, CI 0.78–0.99 and 0.95, CI 0.92–0.99), respectively. Females were more likely to be tested (OR 1.18, CI 1.16–1.21).

The number of lung cancer patients in each HRR and the distance from an NCI cancer center had an inverse relationship on likelihood to be tested. For each additional 100 patients with lung cancer in the HRR, there was a 1.7% decrease in likelihood to be tested. A 50-mile increase in distance from an NCI cancer center decreased the odds to be tested by 5.8%.

## Discussion

This analysis illustrated underutilization of *EGFR* testing. It also demonstrated regional and patient-level differences in access to guideline-recommended lung cancer molecular testing. Based on population-level cancer registry data, guidelines recommend *EGFR* testing for approximately 75,000 patients. We identified 12,433 patients diagnosed and tested in 2013. As illustrated in other studies, Asian women are most likely to be tested, which suggests that physicians’ decisions to test are influenced by the probability of finding a mutation [29]. Analysis of access to testing among most minority groups illustrated that there exists a compounded disparity. Fewer Black and Hispanic patients undergo lung cancer biopsies that produce enough tissue for molecular analysis, which automatically impedes access to tumor tissue analysis. Even among those Black and Hispanic patients who did undergo lung tumor surgical pathology, they were less likely than Asians or Whites to undergo *EGFR* and *KRAS* testing.

Clinical guidelines recommended testing patients who may have specific mutations that can influence the choice of treatment. It has been well documented that *EGFR* mutations are very common in lung cancer patients of East Asian descent (up to 35%) [30], so higher likelihood of testing among Asian patients was expected. In contrast, Black race was a negative predictor. In 2010, there were conflicting reports on the frequency of *EGFR* mutations in Black populations [31]. However, subsequent research discouraged use of patient race in evaluation of ordering a test. Our own research on *EGFR* testing among U.S. veterans indicated that Black veterans were more likely to have an *EGFR* mutation than Whites [11]. Results presented here suggests that unequal access to surgery contributed to differences in testing frequency between White and Black Medicare beneficiaries. This disparity was compounded by differences in direct access to lung tumor molecular testing. Racial disparities in access to lung cancer molecular testing may be decreased as technologies that measure circulating tumor DNA in peripheral blood become commercially available. These tests, referred to as liquid biopsy tests, offer a noninvasive alternative to tissue biopsy for therapeutic decisions and clinical prognosis in patients with lung cancer. During the time of this study, a serum-based proteomics test, brand name VeriStrat was commercially available. This test was validated in clinical trials [32–36], and in 2013 it was approved for Medicare coverage [12, 37]. Among the 2,488 patients who underwent the proteomic test from 2011 to 2013, 257 (10%) of those tested had no lung biopsy or tumor tissue available for *EGFR* testing, which illustrates the capacity for liquid biopsy tests to improve access to lung cancer molecular testing. Analysis of physician uptake demonstrated that the proteomic test significantly influenced therapy recommendations in NSCLC [38].

The observation that oncologists practicing in Boston are most likely to order lung cancer molecular tests was consistent with the fact that *EGFR* mutations conferring responsiveness to EGFR inhibitors were discovered in the Boston-based Harvard Comprehensive Cancer Centers [29, 39, 40]. Harvard’s Massachusetts General Hospital was also the site of the first study demonstrating the effect of the ALK TKI crizotinib [41].

Clinical reasons may explain variation in testing by age and level of comorbidities. A suspected lung cancer diagnosis can be made by a combination of imaging techniques and sputum cytology [5], solid tissue biopsy may not be performed if the patient’s clinical condition or patient’s decision preclude treatment. These factors may account for some patient-level differences in biopsy and testing. Beneficiaries who were enrolled in Medicare prior to age 65 have disabilities, including end-stage renal disease, that may influence treatment decisions. Likewise, beneficiaries over 75 years old may decline treatment for lung cancer. If test results will not influence the treatment decision, there is limited utility of testing.

However, the strongest negative predictor was Medicaid status, which suggests less access to lung cancer molecular testing for low-income beneficiaries. Certain clinical factors that are not reported in claims, such as stage at presentation or smoking status for patients with squamous carcinoma, may explain some of the differences in rates of testing among Medicaid patients.

Our analysis indicated that for a substantial fraction of lung cancer patients, *EGFR* testing was not performed immediately after diagnosis. For some patients, this delay may have corresponded to their progression to the metastatic stage, when testing was recommended, but it also reflected the changes in clinical guidelines for lung cancer during the time studied. Although the evidence linking *EGFR* mutations and responsiveness to TKIs was established in 2004, the specific recommendations for *EGFR* testing were not issued until 2011 by the National Comprehensive Cancer Network (NCCN) and American Society of Clinical Oncology (ASCO). Based on the 2011 ASCO opinion, initial Local Coverage Determinations were made by several Medicare contractors [12]. The first FDA approval of a companion diagnostic test for *EGFR* took place in 2013 [42]. This evolving clinical and regulatory landscape may partially explain why the utilization of *EGFR* testing was low during the time covered by our study.

Our analysis has several limitations related to the data analyzed. Medicare claims provide an opportunity to evaluate health care interventions in a national cohort of patients. However, claims data do not contain clinical information that is relevant to eligibility for *EGFR* testing, such as the date of diagnosis, cancer stage, histological subtype of disease, and tissue availability. We were able to demonstrate that 95% of the patients analyzed represented newly diagnosed cases and we identified those patients with tissue available for analysis through surgical pathology codes. However, other clinical characteristics were not available. To provide comprehensive analysis of precision medicine, claims data need to be reconciled with cancer registry data and patients’ clinical records. However, if we limited our analysis to Surveillance, Epidemiology, and End Results (SEER) linked Medicare data, it would have represented only 28% of the US lung cancer population. Our goal was to provide a population-level analysis.

Until 2013, most molecular tests were billed using methodology-based, stacked codes and there was no direct way to relate the number of codes billed to the number of tests performed. In our investigation of 2011–2012 claims, we treated code 83912 in the combination with diagnosis codes for lung cancer as a proxy for *EGFR*/*KRAS* testing, but this type of analysis can only yield approximate numbers. We believe that our analysis of 2013 claims data is more accurate, as it is based on unique billing codes. Introduction and further expansion of gene-specific Tier1 codes will allow researchers to determine accurately the utilization of specific biomarkers.

## Conclusion

The number of molecularly targeted drugs to treat lung cancer continues to expand, which increases the importance of providing all patients access to molecular testing. In October 2017, FDA awarded breakthrough therapy designation to osimertinib for first-line treatment of patients with metastatic EGFR mutation-positive NSCLC. Only patients with known mutation status will be eligible for osimertinib treatment. Our study demonstrated an overall increase in EGFR testing from 2010 to 2013. However, there was widespread underutilization of guideline-recommended testing. We observed racial, income, and regional disparities in testing. Precision medicine has increased the complexity of cancer diagnosis and treatment. Targeted interventions and clinical decision support tools are needed to ensure that all patients are benefitting from advances in precision medicine. Without such interventions, precision medicine may exacerbate racial disparities in cancer care and health outcomes.

## Additional files

Additional file 1: FDA-Approved Molecularly Targeted Treatments for Lung Cancer. (XLSX 9 kb)

Additional file 2: Timeline of selected clinical guidelines and regulatory decisions. (XLSX 12 kb)

Additional file 3: Number and percentage of lung cancer patients who underwent tumor molecular testing. (XLSX 60 kb)

## Abbreviations

ALK: anaplastic lymphoma kinase
CPT: Current Procedural Terminology
EGFR: epidermal growth factor receptor
FDA: Food & Drug Administration
HCC: Hierarchical Condition Categories
HCPCS: Healthcare Common Procedure Coding System
HRR: Hospital Referral Region
ICD-9: International Classification of Disease
NCI: National Cancer Institute
NSCLC: non-small cell lung cancer
TKI: tyrosine kinase inhibitors
TNBA: transbronchial needle aspiration
US: United States
